# Supplementary material for: Are carbon emissions trading and green financial instruments synergistic? -Comprehensive quantitative research based on content analysis
Source: PLoS One. 2024 Mar 7;19(3):e0298601. doi: 10.1371/journal.pone.0298601 (PMC10919601; doi:10.1371/journal.pone.0298601)
Supplement: S1 Data — (ZIP) [file pone.0298601.s001.zip › Data/地级市Z_F工作报告.zip!/╔╜╢1⁄2/╠⌐░▓╩╨/└┤╫╘╣1⁄2╓┌║┼í╛┬φ┐╦╩2╛▌═°í┐_2018.docx]

各位代表：

　　现在，我代表市人民政府向大会报告工作，请予审议，并请市政协各位委员提出意见。

　　一、2017年政府工作回顾

　　2017年是新一届政府的开局之年。面对艰巨繁重的改革发展稳定任务，在中共泰安市委的坚强领导下，全市上下深入践行五大发展新理念，努力增创五大发展新优势，经济社会发展取得新成绩。

　　（一）经济运行保持平稳向好，转型升级不断加快。全市预计实现地区生产总值3460亿元，增长6.8%左右。完成一般公共预算收入207.1亿元，同口径增长3.9%。完成固定资产投资3100亿元，增长7%左右。实现社会消费品零售总额1600亿元，增长10%左右。城镇和农村居民人均可支配收入分别达到32750元、15650元，增长8.1%和8.5%。三次产业结构调整为8.5∶43.3∶48.2。规模以上工业增加值增长6.5%左右，主营业务收入过亿元的企业达到810家，其中过10亿元的70家。新增中国驰名商标4件、山东名牌产品24个。服务业增加值占生产总值比重提高1.5个百分点，税收增长12.6%，占全部税收比重达到49%。加快构建全域旅游发展新格局，预计接待游客6700万人次，旅游消费总额突破700亿元，分别增长8%左右和12%左右。电子商务交易额突破千亿，达到1050亿元，增长33%。粮食生产保持稳定。蔬菜种植、奶牛存栏、苗木花卉、泰山茶等特色产业有了新发展。规模以上农业龙头企业、农民合作社、家庭农场分别发展到960家、8337家和3938家。省知名农产品区域公用品牌和企业产品品牌达到13个，地理标志证明商标达到39件。休闲农业、观光农业、光伏农业发展迅速。

　　（二）基础设施建设持续加强，城乡面貌不断改观。《泰安市城市总体规划（2011-2020年）（2017年修订）》获批实施。重大基础设施加快建设，以环山路、明堂路、万官大街、桃花源路相连接的中心城区外环线，以泰楼路、一天门大街、新104国道、环山路相连接的城市大环线即将贯通。开工海绵城市项目33个，建成地下管廊10.1公里。泰城新开通和调整公交线路67条。深入开展治理违法建设行动，累计拆违治违97163处、4392万平方米，新增绿化用地面积174万平方米。

　　统筹城乡一体发展，预计常住人口城镇化率提高2个百分点左右。新改建农村公路442公里，全市所有贫困村达到道路通畅标准，具备通车条件的建制村全部通公交车或客车。105国道东平绕城北段改建工程建成通车。投资29亿元，完成水利工程7100项，王家院水库、胜利水库大坝除险加固等工程全面完工。8.9万农村人口饮水安全问题得到解决。岱岳区和新泰市列入国家水土保持重点县，东平县入围全国高效节水示范县。肥城市列入全省中等城市试点，宁阳县列入全省I型小城市试点。满庄镇入选第二批全国特色小镇。2个乡镇、3个村被评为全省第四批美丽宜居小镇、村庄，新增省级美丽乡村28个。大力推进“厕所革命”，完成农村改厕31.02万户。

　　（三）重点领域改革深入推进，开放力度不断加大。圆满完成37项省级以上改革试点任务。供给侧结构性改革扎实推进，“三去一降一补”有效落实。深化国资国企改革，国有资产保值增值率达到108%。现代预算制度主体框架基本确立，市以下财政事权和支出责任划分改革正式启动。预计全市金融机构新增贷款增长8.5%；3家企业在新三板成功挂牌，瑞星集团在香港借壳上市；金融风险得到有效防控化解。事业单位分类改革稳妥推进。综合行政执法体制改革高标准完成。扎实推进农村土地“三权”分置，集体产权制度改革全面铺开。供销社综合改革取得明显成效。首创“河长巡查制度”，市县乡村四级河长组织体系全面建立。探索实行田长制，夯实耕地保护责任。

　　对外开放进一步扩大。预计新批外商投资企业56家，实际使用外资37.54亿元，增长10%。完成进出口总额150亿元，增长11.4%，其中出口116.2亿元，增长7%。对外承包工程合同额增长6.6%，境外实际投资额增长248.2%。岱银集团在马来西亚设立纺织工业园，泰安高新区中德（泰安）安全科技产业园、柬埔寨金泰综合开发区等项目积极推进。

　　（四）民生保障水平较大提高，社会事业不断进步。各项民生财政支出282.2亿元，占一般公共预算支出比重达到79.3%。加大脱贫攻坚力度，落实各级财政专项扶贫资金2.7亿元，实施财政产业扶贫项目459个，预计5.6万人实现稳定脱贫；创新推进东平县易地扶贫搬迁、黄河滩区迁建、移民避险解困“三大工程”，为全省易地扶贫搬迁提供了样本。实施就业优先战略，全年新增城镇就业5.55万人，城镇登记失业率2.35%。新泰市被确定为全国返乡就业试点县。预计全年发放各项社会保险待遇160亿元。城乡低保、特困供养及专项救助水平不断提高，社会救助体系日益完善。我市被确定为国家康复辅助器具产业综合创新试点地区。实施棚户区改造34039套，完成农村危房改造3147户。投入51.89亿元，新改扩建中小学237处，推进解决中小学大班额问题，基本完成“全面改薄”任务。新改扩建幼儿园101处，全面完成学前教育二期三年行动计划。泰安一中新校区建成招生，市特教中心落成启用。现代职业教育体系不断完善。健康泰安加快建设，高端医疗康养产业迅速隆起。新泰市、肥城市通过国家卫生城市复审。稳妥实施全面两孩政策，人口形势保持稳定。深入实施文化惠民工程，公共文化服务体系不断完善；东平县被评为“地名文化千年古县”。深入推进全国文明城市创建，市民文明意识和城市文明程度得到提升。体育事业取得新成绩，泰山国际登山节登泰山比赛，被评为首批国家体育旅游精品赛事。对口援建工作成效明显。国防动员、双拥共建、人民防空、民兵预备役和妇女儿童、老龄工作都有新提高。外事侨务、民族宗教、史志档案等社会事业全面进步。社会治理不断加强，平安建设深入推进，圆满完成党的十九大安保维稳工作。泰山区获全国平安建设先进区称号。食品药品监管能力明显提升。安全生产形势持续稳定。

　　（五）工作推进机制日趋完善，行政能力不断提升。着眼提高履职能力，建立健全“五大工作机制”，各项决策部署得到高效落实。政务督查典型经验在全国政府督查系统交流推广。群众诉求渠道更加畅通，12345热线等民生平台累计受理群众来电42万件，近30万个问题得到解决。坚持依法行政，认真落实全面从严治党要求，坚决贯彻中央八项规定精神，锲而不舍纠“四风”转作风。严格落实“一岗双责”，政府系统廉政建设不断加强。积极推进“两学一做”学习教育常态化制度化，深入开展“重效率、勇担当、作表率”主题活动，健全正向激励和容错纠错机制，大力倡树“不找借口、争创一流”进取精神，形成了比学赶超、争先进位、竞相发展的浓厚氛围。

　　各位代表，2017年是泰安发展历史上极不平凡的一年，是非常重要的一年，也是十分关键的一年。这一年，在市委的领导下，推进完成了几项重大战略性任务。

　　一是重点抓了功能区调整，城市发展空间进一步拓展。立足泰安长远发展，对泰安高新区、泰山景区、泰安旅游经济开发区管辖范围进行调整整合，规划实施“南北两大功能区”“东西两大新城组团”，拉开了城市发展大框架，这是关系全市发展的百年大计。南部，泰安高新区由118平方公里拓展到748平方公里。北部，泰山景区由192平方公里拓展到452平方公里。东部，积极推进济泰同城化，30平方公里的组团式、生态化、节点式新型社区和新型园区正加紧规划。西部，将高铁新区与泰安旅游经济开发区整合，实现了51.2平方公里区域的南北贯通。同时，按照产城融合发展思路，着力抓好京沪高铁以西、新104国道以东约116平方公里的岱岳区新城区规划建设，培育区域核心增长极。

　　二是重点抓了新旧动能转换重大工程，“四新”经济进一步发展壮大。把新旧动能转换作为贯彻新发展理念、深化供给侧结构性改革的重大举措，筛选提报了一批重大政策、工程和项目，纳入国家和省战略规划盘子。康平纳智能染整装备项目入选全省新旧动能转换重大工程项目。深入推动信息化和工业化、制造业与互联网深度融合，泰开集团、石横特钢、泰山玻纤、山能重装、岱银集团、瑞星集团、华鲁锻压等传统行业企业，持续实施技改扩规，延链补链强链，市场竞争力明显增强。泰邦生物、海斯摩尔、晶泰星光电科技、皆瑞金属3D打印、瑞福锂业、诺莱产业园、润德生物等战略性新兴产业不断壮大。峰松物流、润恒物流、泺亨物流、华能能源交通、唯品会仓储物流基地等现代物流业加快发展。泰山云谷、神农智谷、泰山云安智能小镇、跨境电商、农村电商，摩拜和ofo共享单车等数字经济、共享经济蓬勃发展。

　　三是重点抓了工业发展布局，“三强”企业培植力度进一步加大。围绕做大做强工业经济，着力构建“一高三强两突破”工业布局。泰安高新区30平方公里高新高端产业聚集区起步区加快建设，全区国家级高新技术企业达到51家，高新技术产业产值占规模以上工业企业产值的比重达到60.6%，被确定为国家知识产权试点园区。出台了大力培植“三强”企业意见，预计“三强”企业主营业务收入、利润、利税增幅分别高于全市规模以上工业企业14个、31个和42个百分点。新泰市围绕培强做大煤电化、高端装备制造、战略性新兴产业三大产业集群，加快资源枯竭型城市转型发展。肥城市围绕锂电、石墨、盐岩等产业打造产业集群，工业转型升级速度加快。

　　四是重点抓了招商引资体制机制创新，一批重大项目建设进一步加快。调整完善指挥部领导体制和工作机制，将原来十大指挥部整合为五大指挥部；充分发挥泰山国际登山节品牌作用，搭建成果集中展示的大舞台和项目引进签约的大擂台；修订经济社会发展综合考核办法，加大招商引资工作分值权重，对县市区、泰安高新区、泰山景区及市直部门招商工作分别实行“6+2”和分类绩效考核；实行项目春季集中开工和一年两次现场观摩、打分评价，全市抓招商上项目的干劲越来越足、氛围越来越浓，招商引资和重点项目建设发生了质的变化。131个市级重点项目完成投资510亿元，6个省重点项目完成投资62.1亿元。预计到位市外资金336亿元，增长12.5%。泰山康养·高速大成、徂徕山抽水蓄能电站、新泰采煤沉陷区光伏领跑技术基地、阿斯德科技、晋煤明升达洁净煤气化、泰山玻纤产业园、中京生物医药科创园、汉世伟循环农业产业园等一批投资过百亿元项目加快建设。

　　五是重点抓了融资创新园区平台建设，支撑保障措施进一步完善。组建市财金集团，设立产业直投基金。改建新建了国泰民安、金融控股等四大国有资本投资运营公司，走出了一条“国资撬动、市场运作、多元融资、滚动发展”的新路子。泰山创新谷进入实体建设阶段；中研复合材料产业技术研究院、石墨烯技术应用研究院、新泰中关村信息谷创新基地、泰山健康产业园加快建设；国家级工程实验室、企业技术中心分别达到2家和4家，建成国家级工程技术研究中心1家。全市研究与试验发展经费占GDP比重连续3年超过全省平均水平，新认定高新技术企业58家。出台突破园区转型发展意见，园区体制机制逐步顺畅，发展环境日益优化。泰安高新区在全国147个国家级高新区排名中前移8个位次。肥城经济开发区被批准为省级经济开发区。

　　六是重点抓了“放管服”改革和生态文明建设，营商环境进一步优化。出台了“1+4”一揽子“放管服”改革方案，连续10批削减市级行政权力事项509项，将2424项市级行政权力事项授权（委托）泰安高新区行使，授权部门全部刻制“2号章”。在建设领域推行“一窗受理，代办服务”和“容缺预审，绿色通道”。扩展“双随机一公开”覆盖范围，明确了248项随机抽查事项，建立了公共信用信息共享平台。首批公布市县两级154项“零跑腿”和1237项“只跑一次”事项清单。实施“五十证合一、一照一码”改革，事项整合数量位居全省首位。

　　深入推进绿色生态发展，泰山山水林田湖生态保护修复工程成功入选全国试点，我市项目投资达到193亿元，被确定为拉动全省全局工作重大事项和新旧动能转换重大工程。规范河砂资源管理，整顿山石资源开采，加强泰山石、山泉水保护。以中央、省环保督察为契机，抓好环保突出问题整改，关停“散乱污”企业6809个，淘汰燃煤小锅炉2820个，关闭搬迁禁养区内养殖场6467个。实施大气、水、土壤污染防治“三大战役”，主要污染物排放总量持续下降，空气质量稳居全省内陆城市前列，大汶河、东平湖水质达到南水北调要求。实施“绿满泰安”行动，全市森林覆盖率达到39.62%，莲花山国家森林公园被确定为“中国森林氧吧”，5个乡镇、25个村入选全省首批省级森林镇村。大力开展生态示范创建，建成省级以上生态乡镇65个，省级生态村30个。

　　各位代表，工作充满挑战，成绩来之不易。这是新一届市委科学决策、坚强领导的结果，是市人大、市政协及社会各界有效监督、全力支持的结果，凝聚着全市人民的辛劳和智慧，得益于方方面面的支持和奉献。在此，我代表市人民政府，向全市广大干部群众、各民主党派、工商联、无党派人士，向人民解放军和武警驻泰部队及驻泰单位，向关心支持泰安发展的港澳台同胞、海外侨胞及海内外朋友，表示崇高的敬意和衷心的感谢！

　　在看到成绩的同时，必须清醒地看到，我们的工作还面临不少困难和挑战。主要表现在：推动高质量发展还存在一些弱项和短板，传统产业转型升级力度不够大，新兴产业发展速度不够快，企业通过科技创新的内生动力不够强，新旧动能转换任务还很艰巨；投入不足，大项目少，拉动力弱的局面还没有根本改观；实体经济发展还面临诸多困难，融资难、融资贵的问题还没有根本解决；城市综合服务功能还不够完善，城市管理、社会治理还存在薄弱环节，公共服务、民生保障与群众期盼还有差距；干部执政本领和工作作风与新时代新要求还不相适应，行政效能、发展环境还需进一步提升和优化等。面对这些矛盾和问题，需要我们以敢于担当的勇气，采取更加切实有效的措施，付出艰辛的努力，认真加以解决。

　　二、2018年政府工作安排

　　2018年是贯彻党的十九大精神的开局之年，是改革开放40周年，是决胜全面建成小康社会、实施“十三五”规划承上启下的关键一年，做好今年的政府工作意义重大。

　　总体要求是：全面贯彻党的十九大精神，以习近平新时代中国特色社会主义思想为指导，按照中央和省市委决策部署，坚持稳中求进工作总基调，坚持新发展理念，坚持以人民为中心的发展思想，紧扣我国社会主要矛盾变化，按照高质量发展的要求，以“走在前列、位次前移”为目标，以供给侧结构性改革为主线，加快实施新旧动能转换重大工程，统筹推进稳增长、促改革、调结构、惠民生、防风险等各项工作，推动质量变革、效率变革、动力变革，加快推进富裕文明幸福新泰安建设进程。

　　主要预期目标：生产总值增长7%左右；一般公共预算收入增长6%；固定资产投资增长8%左右；社会消费品零售总额增长10%左右；外贸进出口保持稳定增长；实际利用外资增长10%左右；城镇居民人均可支配收入增长8%左右，农村居民人均可支配收入增长8.5%左右；服务业增加值占比提高1.5个百分点左右；战略性新兴产业增加值占生产总值比重提高1个百分点以上；研发投入占GDP比重高于全省平均水平；常住人口城镇化率和户籍人口城镇化率均提高1个百分点左右；全面完成省政府下达的节能减排降碳等约束性指标。

　　（一）加快新旧动能转换，在建设现代化经济体系上实现新突破。借势借力谋划发展全局。山城一体是泰安最大的特色，人文生态是泰安最核心的价值所在，与省会同城是泰安最优越的条件。全市各级必须放远眼光、放宽视野、放大格局，发挥我市“两山一河”人文生态和资源优势，依托正在建设的济泰高速和青兰高速、即将开通运营的济青高铁和北京至上海“复兴号”高铁，以及正在谋划争取的“一山一水一圣人”黄金旅游线城际高铁，巧借济南、青岛、北京、上海的人才、科技、资本和市场等生产力要素，大力发展现代物流、电子商务、会展经济、休闲度假、研学旅游、康养居住、教育培训、文化体育、科技服务和高新高端等特色现代化产业。

　　加快产业转型升级步伐。大力推进供给侧结构性改革，重点在“破立降”上下功夫，实现“三去一降一补”新突破。坚持工业强市和主体支撑作用不动摇，继续大力实施“一高三强两突破”，强力推动泰安高新区高新高端产业集聚，打造新旧动能转换示范区；强力推动“三强”企业培植，支持各县市区构建科学合理、运营良好的梯次企业组织格局；强力突破新泰市、肥城市工业龙头带动作用，实现凤凰涅槃、转型发展。加大优势产业集群培植力度，立足现有基础，规划发展特高压输变电装备、新材料、现代物流、文化旅游等四大千亿产业集群。大力培育发展新能源、节能环保、生物医药、高端装备等战略性新兴产业，做大做强一批拥有自主知识产权、核心竞争力强的行业领军企业。积极推动互联网、大数据、人工智能和制造业深度融合，加快化工、纺织、煤炭、钢铁等传统产业投资、技术、管理创新。深化国有企业改革，完善国有资本授权经营体制，上半年全部纳入国资统一监管体系。积极发展混合所有制经济，推动国有资本做强做优做大。促进非公有制经济发展，培育壮大中小微企业。大力弘扬工匠精神，深入实施质量强市和品牌战略。加大企业家培育培养力度，激发和保护企业家精神。

　　加快实施服务业载体培育工程，着力推进服务业重点行业和领域转型升级。充分发挥旅游经济的王牌牵引作用，进一步强化“大景区、大旅游、大项目、大目标”意识，深入实施“旅游+”战略，推进旅游产业跨界融合发展，全力创建国家全域旅游示范市。围绕“两山一河”，强力推进旅游大项目建设，科学规划、精心实施建设好南北两大景区。整合全市旅游资源，加大旅游营销推广力度，扩大“平安泰山”品牌知名度。推动乡村旅游提档升级，打造“一带三环多片”乡村旅游格局。健全旅游综合执法机制，持续开展旅游环境综合整治，加快旅游厕所、智慧旅游平台、旅游集散中心、旅游标识牌等服务设施建设。

　　实施乡村振兴战略。坚持农业农村优先发展，全力推进城乡统筹和一二三产融合发展。围绕产业兴旺，优化区域布局，着力打造环泰城都市农业圈和沿汶河特色产业带，加快推进现代农业产业园建设，构建现代农业产业新体系。增加绿色优质农产品供给，大力发展现代生态农业、智慧农业、田园综合体等农村新产业新业态。着力构建全市现代水网，加快55座小型水库除险加固、13个大中型水库维修养护和山洪灾害防治等项目建设。围绕生态宜居，深入开展农村人居环境三年整治行动，大力推进“厕所革命”，加快建设美丽乡村。稳步推进农村新型社区建设，全面完成农村社区服务中心建设三年行动计划。建设“四好农村路”，改造农村公路280公里。围绕乡风文明，深入开展乡村文明行动，推动移风易俗、文明进步、崇德向善的淳美乡风。加强特色村居、传统村落保护，挖掘文化内涵，开展镇村历史文化展示工程，留住乡村记忆。围绕治理有效，创新农村社会治理，健全自治、法治、德治治理体系；落实第二轮土地承包到期后再延长30年的政策，让农村更加和谐稳定。围绕生活富裕，深化农村土地“三权”分置制度改革，发展适度规模经营，大力培育新型农业经营主体。全面推进农村集体产权制度改革，持续深化供销社综合改革，保障农民财产权益，支持农民就业创业，不断提高农民收入水平。

　　（二）聚焦关键要素支撑，在增强经济发展内生动力上实现新突破。加大科技创新力度。突出我市区域特色，集聚各类创新资源，创造性地抓好科技研发投入、高新技术产业壮大、科技园区建设等工作。加快构建以泰山创新谷为核心的科技创新体系，完成5万平方米物理空间建设，发挥市科技创新服务云平台作用，推动更多科技成果在泰安转化，形成在全省乃至全国具有品牌影响力的科技创新中心。大力发展高新技术产业，实施高新技术企业和创新型企业培育计划，力争高新技术企业达到160家；落实好省市小微企业创新券管理使用优惠政策，新增科技型中小企业200家以上，力争年底全市高新技术产业产值同比增长10%以上，占规模以上工业总产值比重达到30.8%以上。深化与国内著名高校院所多方位、深层次合作，做强做大一批高水平产业研究院。加大资金政策支持力度，积极推动重大科技成果产业化。创新人才引进使用模式，加大对高层次创新人才、产业领军人才和高技能人才的引进力度，促进人才链、创新链和产业链精准衔接。

　　创新现代投融资模式。坚持“财政+基金+金融”投融资模式，加快政府融资平台市场化转型。扩大产业直投基金规模，设立新旧动能转换基金，支持“四大”优势产业集群、“三强”企业发展。设立文化旅游发展投资基金，支持做强文化旅游产业。设立风险投资基金，支持“双创”、小微种子和上市后备等企业发展。研究设立人才科技基金，支持人才引进、人才培养和高科技产业发展。设立泰山云安智能小镇基金，打造网络信息安全高地。用好80亿元的PPP基金，支持城市、园区基础设施改善和生态文明建设。着力抓好金融服务实体经济，构建多层次、多样化、覆盖全市各类企业的政金企合作体系。积极开展“资本市场年”活动，加快规模企业规范化公司制改制，推动企业上市挂牌，不断扩大直接融资规模。发展壮大泰安银行、农商银行等地方金融机构。积极推进大宗商品交易平台建设，形成辐射全国的大宗商品交易中心。

　　加快园区载体建设。充分发挥园区聚集生产要素、推进产业转型升级、扩大对外开放的重大载体作用，坚持园区建设与城市发展走向一致、与产业布局高度契合原则，持续完善管理体制、用人分配机制和市场运作机制，加快各级各类园区功能整合和转型发展。鼓励园区积极承接外资项目，大力引进世界500强和跨国企业集团，着力培育壮大外贸进出口企业，整体提升园区的发展活力和开放水平。加大对园区的考核力度，促进各级各类园区不断发展壮大，实现位次前移。支持新泰经济开发区、肥城高新技术产业开发区争创国家级园区。

　　大力优化营商环境。深入推进“放管服”改革，继续取消下放一批行政许可等权力事项，全面推进“互联网+政务服务”、“双随机一公开”监管和政务服务大厅“一窗受理，集成服务”，试点推进“证照分离”，加快社会信用体系建设，努力打造审批事项少、办事效率高、服务质量优和诚实守信的服务环境。

　　（三）着力提升城市功能品质，在城市转型发展上实现新突破。城市发展举足轻重，必须充分发挥城市经济的龙头带动作用，强化城市意识，尊重城市发展规律，走“环境-人才-科技-产业”内涵发展之路，全面提升城市发展水平。

　　进一步加快功能区建设。强化科学规划理念，高水平开展空间发展战略研究。加快泰城南北两大功能区和东西两大新城组团规划建设，着力打造“北依泰山、南接徂徕、汶水环绕、山城一体”的现代化生态宜居名城。南部，着力发展高端装备制造业和战略性新兴产业，规划建设八大特色产业园，构建“一区多园”发展格局。北部，加大泰山核心景区保护提升，加快建设以“泰山慢谷”为主题的大景区。东部，加快建成集旅游休闲、户外运动、科技研发、高端制造、生态绿地为一体的现代化产业新城。西部，加速泰安旅游经济开发区、高铁新区的融合升级改造，打造旅游服务、总部办公、高端居住等为一体的现代化宜居新城。

　　进一步完善配套设施。加大济泰高速、青兰高速泰城段升级、京台高速拓宽改造和泰山西出口建设的推进力度，加快形成泰城高速外环线；加大泰楼路拓宽升级建设的推进力度，加快形成贯穿泰山景区、泰山区、徂汶景区南北三区的大通道。加快推进市垃圾处理厂和第四污水处理厂扩容改造工程。积极推进引黄入泰工程，开工建设泰城应急饮用水源地和泰城西部供热中心项目。重启范镇60万吨LNG天然气储备项目，缓解我市气源不足、供求结构性矛盾。抢抓国家棚户区改造政策机遇“窗口期”，年内完成棚户区改造57569户、老旧小区改造8595户，力争3年内全市棚户区和老旧小区全部完成改造。完善城市管理体系，加快智慧城市建设，健全智能交通、智能管网、智能城管、智能安全保障体系。加大城市交通治堵力度，不断优化道路通行环境。强化城市综合执法，加大城市违法建设治理力度。高度重视城市社区建设，不断提高社区服务水平。

　　（四）围绕优势主导产业集群发展，在招商引资和项目建设上实现新突破。坚持把招商引资和项目建设作为转型发展的总抓手，以增量带动存量优化、总量扩张、质量提升，不断增强经济发展后劲。充分发挥泰山国际登山节平台作用，形成“年初下达任务、春季集中开工、季度调度督导、年中年末考核、全年跟踪督查”的闭环招商机制。强化招商引资指挥部资源整合、整体作战能力，进一步完善考核体系，最大限度调动全市各级抓招商上项目的积极性和主动性。转变招商理念，创新招商方式，强化配套服务，进一步发挥企业和园区的招商主体、载体作用，大力实施产业招商、定向招商、精准招商和招才引智，下大气力抓好一批扩大产业影响、促进产业集聚、引领产业发展的“三强”企业，一批“四新”“四化”重大产业项目,一批能够促进质量变革、效率变革、动力变革重大科技创新项目,一批关系泰安长远布局、长远影响、长远发展的重大基础设施工程,一批有突出特色、有强大活力、有明显竞争力的重要发展载体和平台。

　　密切关注国家重点扶持领域和产业发展方向政策措施，精心策划100个前期推进项目，筛选确定130个左右市级重点建设项目，争取更多项目、资金和政策落户泰安。着力抓好徂徕山抽水蓄能电站、泰山慢谷、新泰采煤沉陷区光伏领跑技术基地二期等项目建设。加快推进在建的青兰高速泰安至东阿段、宁梁高速、京沪高速新泰段改扩建等工程；积极推进青兰高速莱芜至泰安段、董梁高速新泰至宁阳段、新泰至台儿庄高速等项目前期工作。争取东平湖湖区航道完工，大清河航道和老湖、银山作业区开工建设。

　　（五）牢记绿水青山就是金山银山，在生态环境建设上实现新突破。高水平、高标准搞好生态文明规划建设，全面提升以“文化生态”为内涵的城市核心竞争力。

　　全力以赴搞好生态保护修复。加快实施泰山山水林田湖生态保护修复试点工程，努力打造“生态友好、环境美丽、功能完善、文化永续”的生态功能区。科学划定城镇、农业、生态空间以及生态保护红线、永久基本农田、城镇开发边界，建立健全主体功能区差异化协调发展长效机制。深入落实最严格水资源管理制度，加快水生态文明建设。

　　坚决打赢污染治理攻坚战。大力开展“蓝天碧水净地”三大行动，巩固扩大环保督察和环保攻坚工作成果。加大燃煤污染、工业无组织排放、城市扬尘、汽车尾气等整治力度，确保各项污染物浓度持续下降、蓝天白云天数持续增加。加大水污染防治力度，实施水源地保护区综合整治，实现大汶河上游支流水质逐步改善。严格保护耕地，加大土壤污染防治力度。

　　坚持不懈抓好造林绿化。继续实施“绿满泰安”行动，集中抓好城区绿色空间、荒山绿化攻坚、绿色廊道、森林镇村、河湖湿地景观林、破损山体植被恢复“六大生态绿化工程”。注重城市绿化景观节点设计，突出“山水石松竹”五大元素，努力打造城区重要路段和窗口区域的园林景观。抓好公路、铁路、河流“三边”生态廊道和大汶河水系生态林带建设。加快荒山绿化进程，力争3年基本实现宜林荒山绿化全覆盖。集中连片实施农田林网建设，打造一批高标准农田林网示范区。

　　（六）坚持以人民为中心的发展思想，在民生保障和社会治理上实现新突破。坚持尽力而为、量力而行，努力在发展中补齐民生短板，不断增强人民群众获得感、幸福感、安全感。

　　坚决打赢精准脱贫攻坚战。坚持精准方略，瞄准特定贫困群众精准帮扶，向深度贫困区域聚焦发力，实施贫困村提升、富民产业培育、社会保障兜底等行动，抓好东平县易地扶贫搬迁、黄河滩区迁建、移民避险解困“三大工程”，确保贫困群众搬得出、稳得住、逐步能致富。深化大扶贫格局，扶贫同扶志、扶智相结合，激发贫困人口内生动力，建立健全稳定脱贫长效机制，确保高质量完成脱贫攻坚各项任务。

　　进一步提高社会保障水平。落实好就业创业政策，抓好大中专毕业生、返乡农民工、就业困难人员等重点群体就业工作，年内确保城镇新增就业4.5万人以上，城镇登记失业率控制在3.5%以内。继续推进全民参保工作，将符合条件的各类群体全部纳入社会保障制度。完善社会保险政策体系，积极试行职工长期护理保险制度。深化医保支付方式改革，强化医保基金监管。进一步提高城乡低保、农村特困供养标准，加强医疗救助、临时救助。积极推进养老事业、养老产业发展。

　　大力繁荣发展社会事业。坚持教育优先发展，加大教育投入，启动义务教育优质均衡发展创建，办好学前教育、义务教育、特殊教育和高中教育，努力让每个孩子都能享有公平而有质量的教育；完善职业教育，深化产教融合、校企合作；着力解决中小学大班额突出问题和“全面改薄”工作任务；保障进城务工人员随迁子女平等接受义务教育，做好家庭经济困难学生资助工作；大力发展民办教育；支持驻泰高校做大做强，深入推进校地融合发展。持续深化医药卫生体制改革，加快健全分级诊疗制度、优质高效医疗卫生服务体系和现代医院管理制度；加强基层医疗卫生服务体系和全科医生队伍建设；稳妥实施全面两孩政策；加强重大疾病综合防治，促进中医药事业健康发展。积极弘扬泰山文化；健全现代公共文化服务体系，深入实施文化惠民工程；持续开展“全民阅读”活动，建设书香泰安；加快泰山出版小镇、泰山博物院建设，创建泰山文化生态保护区；加强文物保护利用和文化遗产保护传承。加大全国文明城市创建工作力度。大力发展体育事业和体育产业。依法管理宗教事务，增进民族团结。认真抓好全民国防教育、国防动员和退役军人管理保障工作。关心支持老龄、妇女儿童、残疾人事业发展。繁荣发展其他各项社会事业。

　　坚决打赢风险防控攻坚战。强化社会治安风险防控，深入推进平安泰安建设，着力抓好矛盾纠纷排查化解和治安要素管控，完善立体化社会治安防控体系，夯实基层基础。强化安全生产风险防控，推进“安如泰山”科学预防体系和预防重特大事故试点，打好化工产业安全生产转型升级攻坚战。强化食品药品安全风险防控，确保群众饮食用药安全。强化金融风险防控，建立全域金融风险防控体系，全力防范化解企业资金链风险，严厉打击恶意逃废金融债务行为。强化政府债务风险防控，建立政府债务风险防控机制。强化应急安全风险防控，加强应急管理和处置能力建设，确保社会和谐稳定。

　　三、全面加强政府自身建设

　　新时代、新目标、新任务，对政府自身建设提出了新的更高要求。我们必须不忘初心再出发，勇担重任立潮头，努力开创政府各项工作新局面。

　　一是强化政治建设，增强凝聚力。始终把抓好政府系统党的建设作为第一责任和最大政绩，切实增强“四个意识”，自觉在政治立场、政治方向、政治原则、政治道路上同以习近平同志为核心的党中央保持高度一致，坚决维护党中央权威和集中统一领导，坚决贯彻落实党中央国务院、省委省政府和市委各项决策部署。把严守政治纪律和政治规矩放在首位，知敬畏、存戒惧、守底线，始终做政治上的明白人。

　　二是强化本领建设，增强创新力。适应新时代新要求，全面系统地学习习近平新时代中国特色社会主义思想，真正领会内涵要义和精神实质，增强行动自觉。牢牢把握发展第一要务，围绕推动高质量发展谋划思路举措，善于结合实际创造性地用好机遇、推进改革、解决问题。学好用好大数据，善于运用互联网技术和信息化手段开展工作。更加注重培养政府系统党员干部的专业能力、专业精神，鼓励更多的优秀干部投身招商引资主阵地、园区建设主战场、民生改善最前沿，练就新时代新作为的过硬本领。健全完善正向激励、考核评价、容错纠错、澄清保护机制，旗帜鲜明为敢于担当、踏实做事、不谋私利的干部撑腰鼓劲。

　　三是强化法治建设，增强公信力。坚持依法行政、严格执法、全面执法，带头尊法学法守法用法，自觉运用法治思维和法治方式推动工作。健全完善执法责任、效能监察、政务公开、举报投诉、复议应诉制度，确保权力依法公正高效运行，始终接受监督。坚持向人大报告工作和向政协通报工作制度，认真执行人大及其常委会的决议、决定，自觉接受人大的法律监督和政协的民主监督，认真办理人大代表议案、建议和政协提案。加强同各民主党派、工商联、人民团体和社会各界人士的联系。推进依法科学民主决策，认真落实重大行政决策法定程序，严格行政决策责任追究，提高决策科学化、民主化、法治化水平。

　　四是强化作风建设，增强执行力。巩固“两学一做”常态化制度化学习教育成果，深入开展“不忘初心、牢记使命”主题教育，驰而不息反对和纠正“四风”，尤其大力整治形式主义、官僚主义，防止“四风”问题反弹回潮。大力弘扬担当之风，以功成不必在我、建功必须有我的胸怀，讲大局、讲奉献、讲责任，争做改革发展的表率。大力弘扬实干之风，紧紧围绕全市重点难点工作，始终做到心中有数、心中有责，尽职尽责、务求实效，争做实干落实的表率。大力弘扬为民之风，扎实推进12345便民服务热线和媒体民生平台互联互动，让群众每一项诉求都在阳光下操作，以实际行动取信于民，争做勤政为民的表率。

　　五是强化廉政建设，增强约束力。落实全面从严治党主体责任，切实履行“一岗双责”，不断完善惩治和预防腐败体系，推动政府系统全面从严治党向纵深发展。坚决支持国家监察体制改革，实现对所有行使公权力公职人员监察全覆盖。加大审计监督力度，强化执纪问责，突出抓好扶贫领域腐败和作风问题专项治理，严惩任何形式的贪污腐败、徇私枉法等违纪违法行为。严格执行廉洁自律准则和纪律处分条例，严格落实中央八项规定实施细则，始终做到一身正气、一尘不染，以清廉政风赢得广大人民群众的信任和支持。

　　各位代表，逐梦之旅无坦途，破浪前行须奋进。让我们紧密团结在以习近平同志为核心的党中央周围，在中共泰安市委的坚强领导下，带着对事业的激情，带着对家乡的感情，带着对人民的深情，不忘初心，砥砺前行，为加快建设富裕文明幸福新泰安而努力奋斗！
